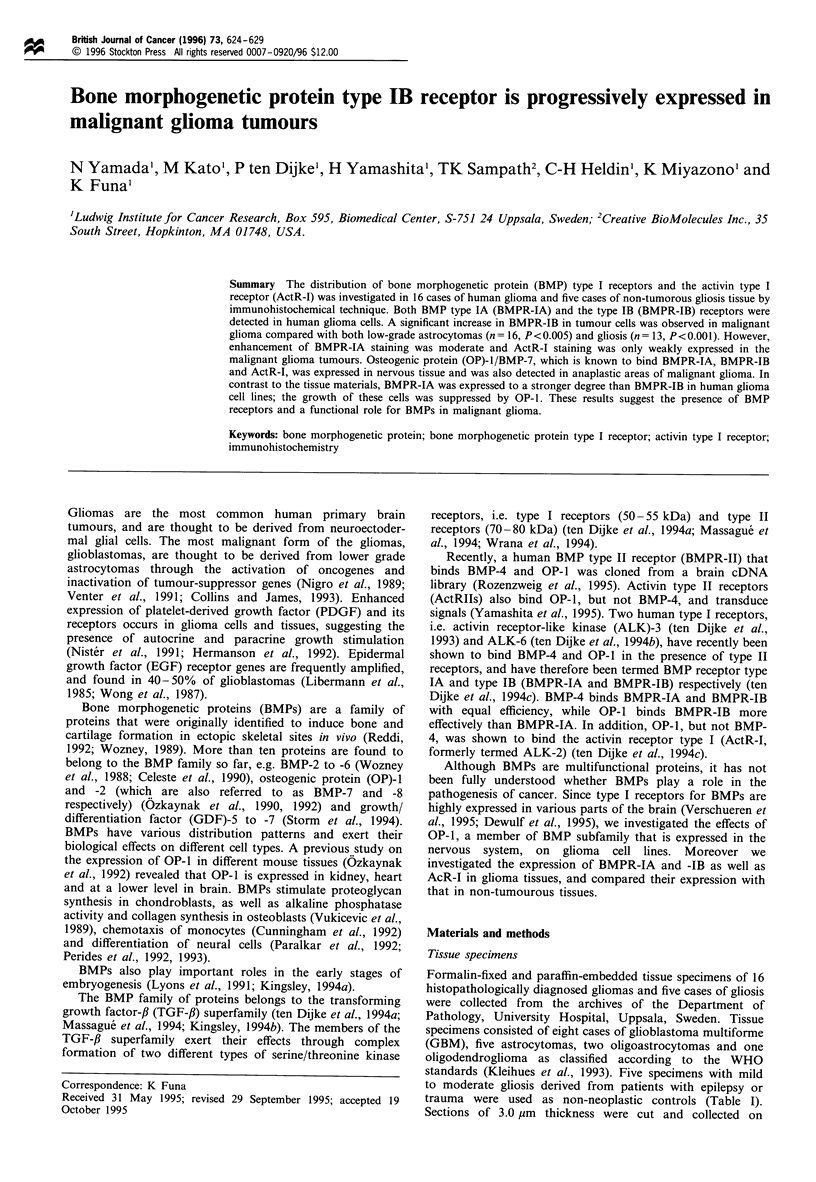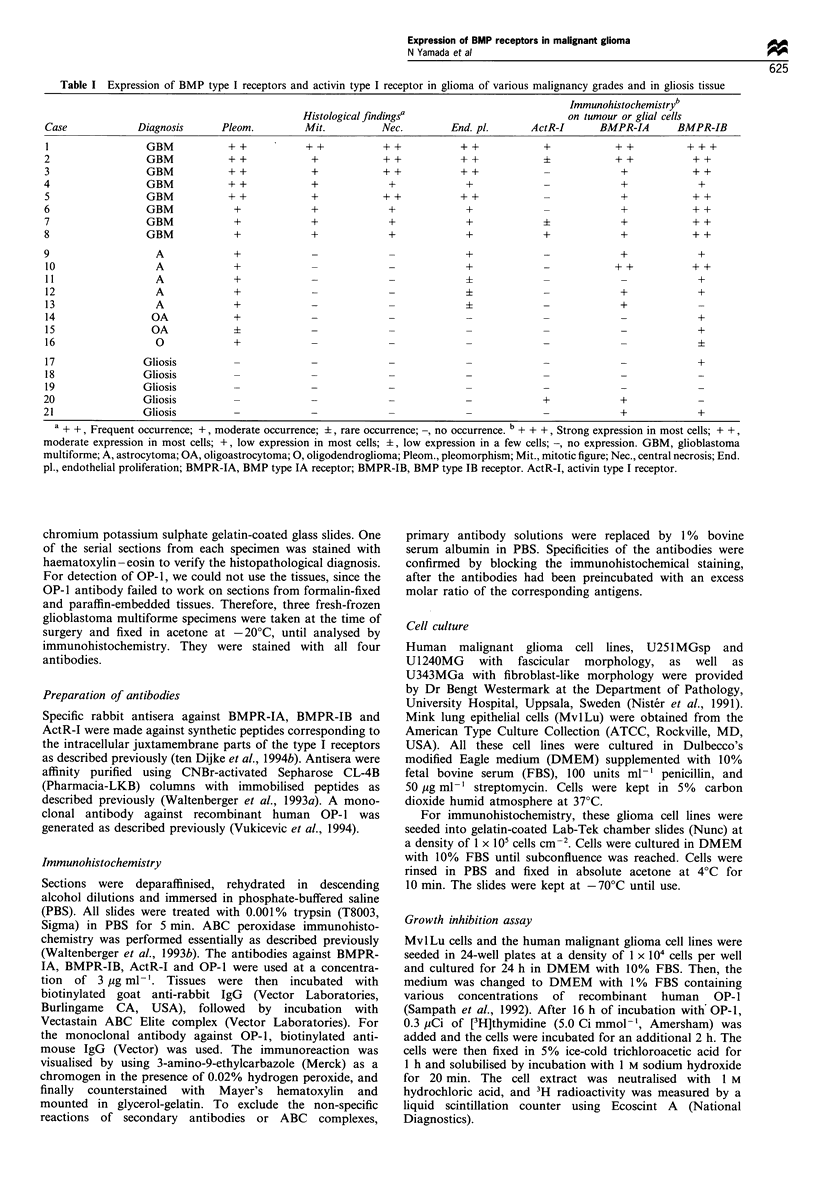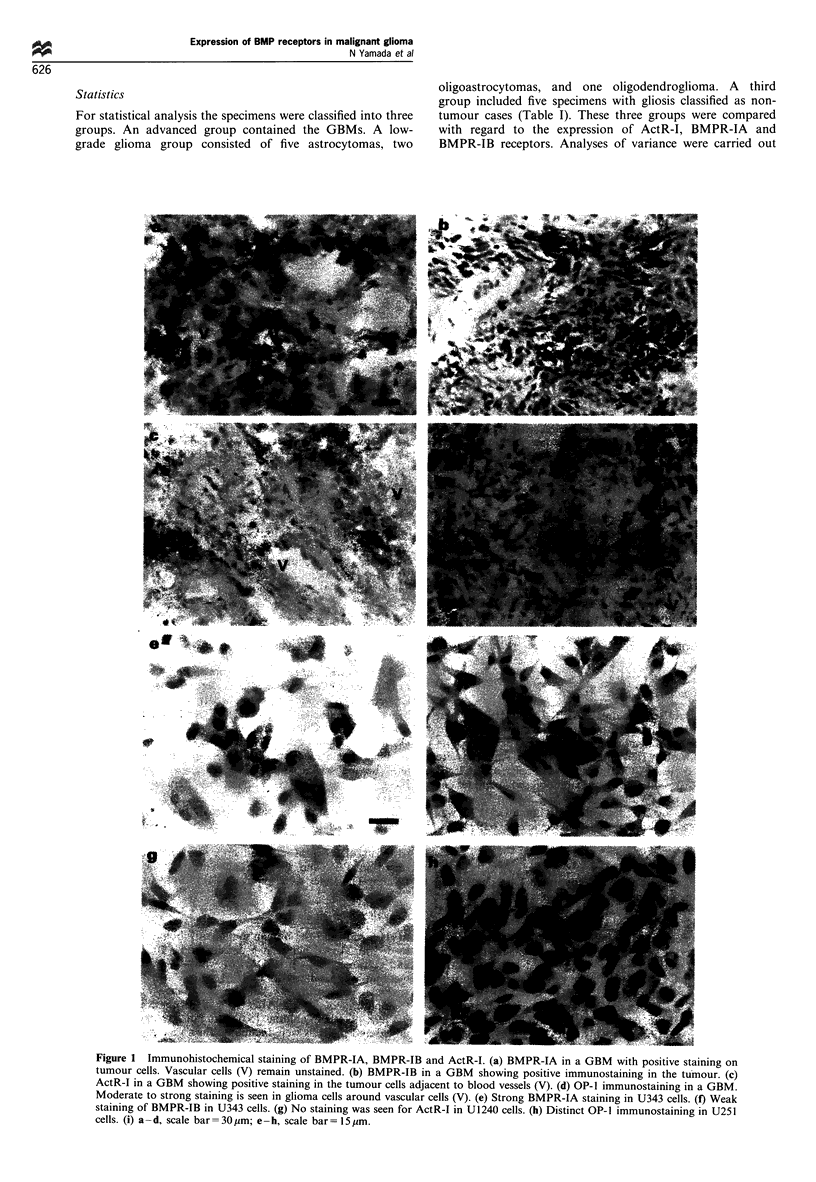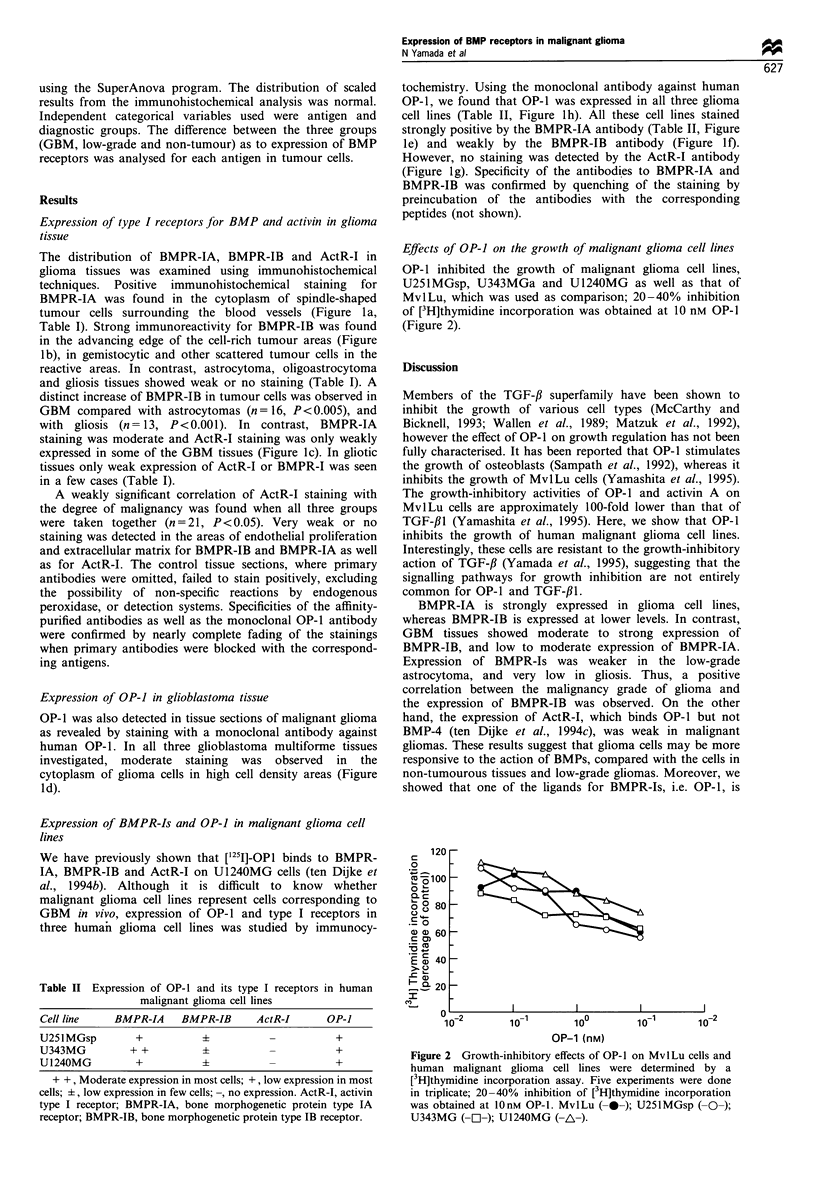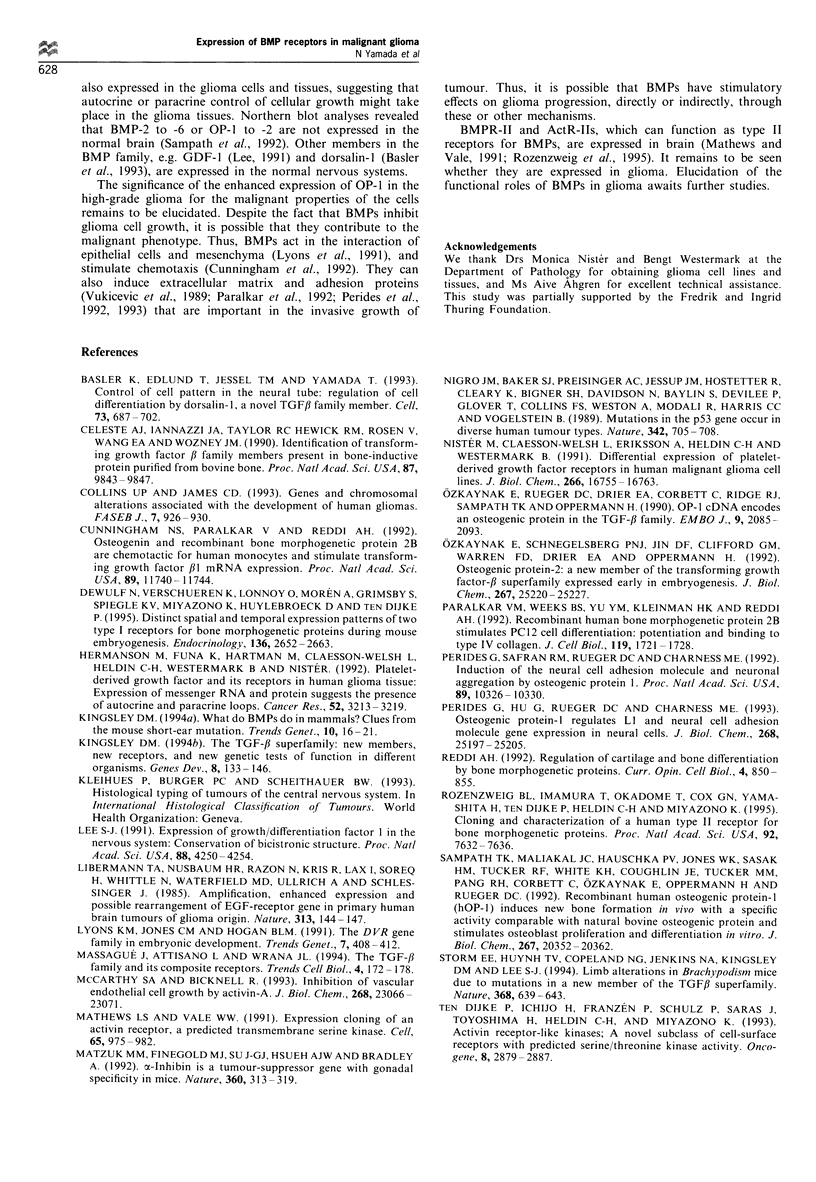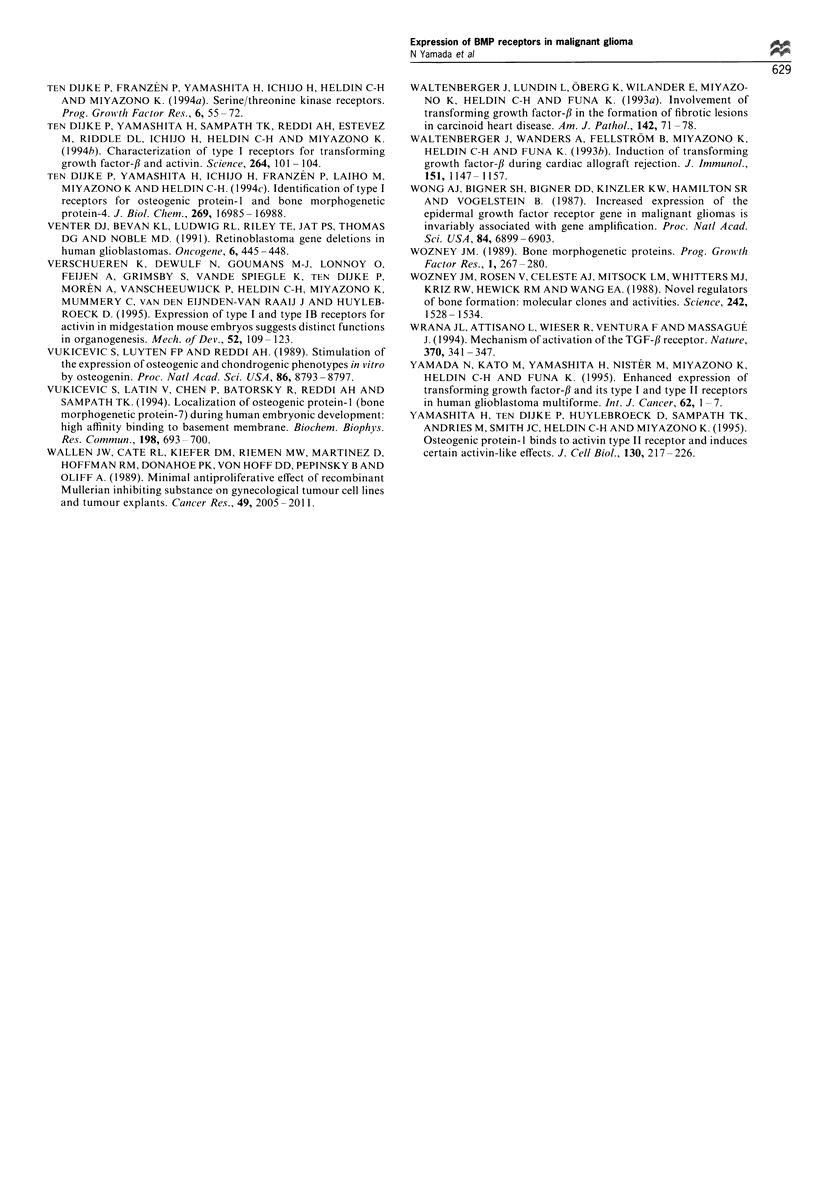# Bone morphogenetic protein type IB receptor is progressively expressed in malignant glioma tumours.

**DOI:** 10.1038/bjc.1996.108

**Published:** 1996-03

**Authors:** N. Yamada, M. Kato, P. ten Dijke, H. Yamashita, T. K. Sampath, C. H. Heldin, K. Miyazono, K. Funa

**Affiliations:** Ludwig Institute for Cancer Research, Uppsala, Sweden.

## Abstract

**Images:**


British Journal of Cancer (1996) 73, 624–629
© 1996 Stockton Press All rights reserved 0007–0920/96 $12.00

### Bone morphogenetic protein type IB receptor is progressively expressed in malignant glioma tumours

N Yamada[1], M Kato[1], P ten Dijke[1], H Yamashita[1], TK Sampath[2], C-H Heldin[1], K Miyazono[1] and K Funa[1]

[1]*Ludwig Institute for Cancer Research, Box 595, Biomedical Center, S-751 24 Uppsala, Sweden;* [2]*Creative BioMolecules Inc., 35 South Street, Hopkinton, MA 01748, USA.*

**Summary** The distribution of bone morphogenetic protein (BMP) type I receptors and the activin type I receptor (ActR-I) was investigated in 16 cases of human glioma and five cases of non-tumorous gliosis tissue by immunohistochemical technique. Both BMP type IA (BMPR-IA) and the type IB (BMPR-IB) receptors were detected in human glioma cells. A significant increase in BMPR-IB in tumour cells was observed in malignant glioma compared with both low-grade astrocytomas ($n=16$, $P<0.005$) and gliosis ($n=13$, $P<0.001$). However, enhancement of BMPR-IA staining was moderate and ActR-I staining was only weakly expressed in the malignant glioma tumours. Osteogenic protein (OP)-1/BMP-7, which is known to bind BMPR-IA, BMPR-IB and ActR-I, was expressed in nervous tissue and was also detected in anaplastic areas of malignant glioma. In contrast to the tissue materials, BMPR-IA was expressed to a stronger degree than BMPR-IB in human glioma cell lines; the growth of these cells was suppressed by OP-1. These results suggest the presence of BMP receptors and a functional role for BMPs in malignant glioma.

**Keywords:** bone morphogenetic protein; bone morphogenetic protein type I receptor; activin type I receptor; immunohistochemistry

Gliomas are the most common human primary brain tumours, and are thought to be derived from neuroectodermal glial cells. The most malignant form of the gliomas, glioblastomas, are thought to be derived from lower grade astrocytomas through the activation of oncogenes and inactivation of tumour-suppressor genes (Nigro *et al.*, 1989; Venter *et al.*, 1991; Collins and James, 1993). Enhanced expression of platelet-derived growth factor (PDGF) and its receptors occurs in glioma cells and tissues, suggesting the presence of autocrine and paracrine growth stimulation (Nistér *et al.*, 1991; Hermanson *et al.*, 1992). Epidermal growth factor (EGF) receptor genes are frequently amplified, and found in 40–50% of glioblastomas (Libermann *et al.*, 1985; Wong *et al.*, 1987).

Bone morphogenetic proteins (BMPs) are a family of proteins that were originally identified to induce bone and cartilage formation in ectopic skeletal sites *in vivo* (Reddi, 1992; Wozney, 1989). More than ten proteins are found to belong to the BMP family so far, e.g. BMP-2 to -6 (Wozney *et al.*, 1988; Celeste *et al.*, 1990), osteogenic protein (OP)-1 and -2 (which are also referred to as BMP-7 and -8 respectively) (Özkaynak *et al.*, 1990, 1992) and growth/differentiation factor (GDF)-5 to -7 (Storm *et al.*, 1994). BMPs have various distribution patterns and exert their biological effects on different cell types. A previous study on the expression of OP-1 in different mouse tissues (Özkaynak *et al.*, 1992) revealed that OP-1 is expressed in kidney, heart and at a lower level in brain. BMPs stimulate proteoglycan synthesis in chondroblasts, as well as alkaline phosphatase activity and collagen synthesis in osteoblasts (Vukicevic *et al.*, 1989), chemotaxis of monocytes (Cunningham *et al.*, 1992) and differentiation of neural cells (Paralkar *et al.*, 1992; Perides *et al.*, 1992, 1993).

BMPs also play important roles in the early stages of embryogenesis (Lyons *et al.*, 1991; Kingsley, 1994*a*).

The BMP family of proteins belongs to the transforming growth factor-$\beta$ (TGF-$\beta$) superfamily (ten Dijke *et al.*, 1994*a*; Massagué *et al.*, 1994; Kingsley, 1994*b*). The members of the TGF-$\beta$ superfamily exert their effects through complex formation of two different types of serine/threonine kinase receptors, i.e. type I receptors (50–55 kDa) and type II receptors (70–80 kDa) (ten Dijke *et al.*, 1994*a*; Massagué *et al.*, 1994; Wrana *et al.*, 1994).

Recently, a human BMP type II receptor (BMPR-II) that binds BMP-4 and OP-1 was cloned from a brain cDNA library (Rozenzweig *et al.*, 1995). Activin type II receptors (ActRIIs) also bind OP-1, but not BMP-4, and transduce signals (Yamashita *et al.*, 1995). Two human type I receptors, i.e. activin receptor-like kinase (ALK)-3 (ten Dijke *et al.*, 1993) and ALK-6 (ten Dijke *et al.*, 1994*b*), have recently been shown to bind BMP-4 and OP-1 in the presence of type II receptors, and have therefore been termed BMP receptor type IA and type IB (BMPR-IA and BMPR-IB) respectively (ten Dijke *et al.*, 1994*c*). BMP-4 binds BMPR-IA and BMPR-IB with equal efficiency, while OP-1 binds BMPR-IB more effectively than BMPR-IA. In addition, OP-1, but not BMP-4, was shown to bind the activin receptor type I (ActR-I, formerly termed ALK-2) (ten Dijke *et al.*, 1994*c*).

Although BMPs are multifunctional proteins, it has not been fully understood whether BMPs play a role in the pathogenesis of cancer. Since type I receptors for BMPs are highly expressed in various parts of the brain (Verschueren *et al.*, 1995; Dewulf *et al.*, 1995), we investigated the effects of OP-1, a member of BMP subfamily that is expressed in the nervous system, on glioma cell lines. Moreover we investigated the expression of BMPR-IA and -IB as well as AcR-I in glioma tissues, and compared their expression with that in non-tumourous tissues.

### Materials and methods

### *Tissue specimens*

Formalin-fixed and paraffin-embedded tissue specimens of 16 histopathologically diagnosed gliomas and five cases of gliosis were collected from the archives of the Department of Pathology, University Hospital, Uppsala, Sweden. Tissue specimens consisted of eight cases of glioblastoma multiforme (GBM), five astrocytomas, two oligoastrocytomas and one oligodendroglioma as classified according to the WHO standards (Kleihues *et al.*, 1993). Five specimens with mild to moderate gliosis derived from patients with epilepsy or trauma were used as non-neoplastic controls (Table I). Sections of 3.0 $\mu$m thickness were cut and collected on

Correspondence: K Funa
Received 31 May 1995; revised 29 September 1995; accepted 19 October 1995

**Table I** Expression of BMP type I receptors and activin type I receptor in glioma of various malignancy grades and in gliosis tissue

| Case | Diagnosis | Histological findings[a] | | | | Immunohistochemistry[b] on tumour or glial cells | | |
|---|---|---|---|---|---|---|---|---|
| | | *Pleom.* | *Mit.* | *Nec.* | *End. pl.* | *ActR-I* | *BMPR-IA* | *BMPR-IB* |
| 1 | GBM | ++ | ++ | ++ | ++ | + | ++ | +++ |
| 2 | GBM | ++ | + | ++ | ++ | ± | ++ | ++ |
| 3 | GBM | ++ | + | ++ | ++ | – | + | ++ |
| 4 | GBM | ++ | + | + | + | – | + | + |
| 5 | GBM | ++ | + | ++ | ++ | – | + | ++ |
| 6 | GBM | + | + | + | + | – | + | ++ |
| 7 | GBM | + | + | + | + | ± | + | ++ |
| 8 | GBM | + | + | + | + | + | + | ++ |
| 9 | A | + | – | – | + | – | + | + |
| 10 | A | + | – | – | + | – | ++ | ++ |
| 11 | A | + | – | – | ± | – | – | + |
| 12 | A | + | – | – | ± | – | + | + |
| 13 | A | + | – | – | ± | – | + | – |
| 14 | OA | + | – | – | – | – | – | + |
| 15 | OA | ± | – | – | – | – | – | + |
| 16 | O | + | – | – | – | – | – | ± |
| 17 | Gliosis | – | – | – | – | – | – | + |
| 18 | Gliosis | – | – | – | – | – | – | – |
| 19 | Gliosis | – | – | – | – | – | – | – |
| 20 | Gliosis | – | – | – | – | + | + | – |
| 21 | Gliosis | – | – | – | – | – | + | + |

[a] ++, Frequent occurrence; +, moderate occurrence; ±, rare occurrence; –, no occurrence. [b] +++, Strong expression in most cells; ++, moderate expression in most cells; +, low expression in most cells; ±, low expression in a few cells; –, no expression. GBM, glioblastoma multiforme; A, astrocytoma; OA, oligoastrocytoma; O, oligodendroglioma; Pleom., pleomorphism; Mit., mitotic figure; Nec., central necrosis; End. pl., endothelial proliferation; BMPR-IA, BMP type IA receptor; BMPR-IB, BMP type IB receptor. ActR-I, activin type I receptor.

chromium potassium sulphate gelatin-coated glass slides. One of the serial sections from each specimen was stained with haematoxylin–eosin to verify the histopathological diagnosis. For detection of OP-1, we could not use the tissues, since the OP-1 antibody failed to work on sections from formalin-fixed and paraffin-embedded tissues. Therefore, three fresh-frozen glioblastoma multiforme specimens were taken at the time of surgery and fixed in acetone at −20°C, until analysed by immunohistochemistry. They were stained with all four antibodies.

### *Preparation of antibodies*

Specific rabbit antisera against BMPR-IA, BMPR-IB and ActR-I were made against synthetic peptides corresponding to the intracellular juxtamembrane parts of the type I receptors as described previously (ten Dijke *et al.*, 1994*b*). Antisera were affinity purified using CNBr-activated Sepharose CL-4B (Pharmacia-LKB) columns with immobilised peptides as described previously (Waltenberger *et al.*, 1993*a*). A monoclonal antibody against recombinant human OP-1 was generated as described previously (Vukicevic *et al.*, 1994).

### *Immunohistochemistry*

Sections were deparaffinised, rehydrated in descending alcohol dilutions and immersed in phosphate-buffered saline (PBS). All slides were treated with 0.001% trypsin (T8003, Sigma) in PBS for 5 min. ABC peroxidase immunohistochemistry was performed essentially as described previously (Waltenberger *et al.*, 1993*b*). The antibodies against BMPR-IA, BMPR-IB, ActR-I and OP-1 were used at a concentration of 3 $\mu g\ ml^{-1}$. Tissues were then incubated with biotinylated goat anti-rabbit IgG (Vector Laboratories, Burlingame CA, USA), followed by incubation with Vectastain ABC Elite complex (Vector Laboratories). For the monoclonal antibody against OP-1, biotinylated anti-mouse IgG (Vector) was used. The immunoreaction was visualised by using 3-amino-9-ethylcarbazole (Merck) as a chromogen in the presence of 0.02% hydrogen peroxide, and finally counterstained with Mayer's hematoxylin and mounted in glycerol-gelatin. To exclude the non-specific reactions of secondary antibodies or ABC complexes, primary antibody solutions were replaced by 1% bovine serum albumin in PBS. Specificities of the antibodies were confirmed by blocking the immunohistochemical staining, after the antibodies had been preincubated with an excess molar ratio of the corresponding antigens.

### *Cell culture*

Human malignant glioma cell lines, U251MGsp and U1240MG with fascicular morphology, as well as U343MGa with fibroblast-like morphology were provided by Dr Bengt Westermark at the Department of Pathology, University Hospital, Uppsala, Sweden (Nistér *et al.*, 1991). Mink lung epithelial cells (Mv1Lu) were obtained from the American Type Culture Collection (ATCC, Rockville, MD, USA). All these cell lines were cultured in Dulbecco's modified Eagle medium (DMEM) supplemented with 10% fetal bovine serum (FBS), 100 units $ml^{-1}$ penicillin, and 50 $\mu g\ ml^{-1}$ streptomycin. Cells were kept in 5% carbon dioxide humid atmosphere at 37°C.

For immunohistochemistry, these glioma cell lines were seeded into gelatin-coated Lab-Tek chamber slides (Nunc) at a density of $1 \times 10^5$ cells $cm^{-2}$. Cells were cultured in DMEM with 10% FBS until subconfluence was reached. Cells were rinsed in PBS and fixed in absolute acetone at 4°C for 10 min. The slides were kept at −70°C until use.

### *Growth inhibition assay*

Mv1Lu cells and the human malignant glioma cell lines were seeded in 24-well plates at a density of $1 \times 10^4$ cells per well and cultured for 24 h in DMEM with 10% FBS. Then, the medium was changed to DMEM with 1% FBS containing various concentrations of recombinant human OP-1 (Sampath *et al.*, 1992). After 16 h of incubation with OP-1, 0.3 $\mu Ci$ of [$^3H$]thymidine (5.0 Ci $mmol^{-1}$, Amersham) was added and the cells were incubated for an additional 2 h. The cells were then fixed in 5% ice-cold trichloroacetic acid for 1 h and solubilised by incubation with 1 M sodium hydroxide for 20 min. The cell extract was neutralised with 1 M hydrochloric acid, and $^3H$ radioactivity was measured by a liquid scintillation counter using Ecoscint A (National Diagnostics).

*Statistics*

For statistical analysis the specimens were classified into three groups. An advanced group contained the GBMs. A low-grade glioma group consisted of five astrocytomas, two oligoastrocytomas, and one oligodendroglioma. A third group included five specimens with gliosis classified as non-tumour cases (Table I). These three groups were compared with regard to the expression of ActR-I, BMPR-IA and BMPR-IB receptors. Analyses of variance were carried out

**Figure 1** Immunohistochemical staining of BMPR-IA, BMPR-IB and ActR-I. (**a**) BMPR-IA in a GBM with positive staining on tumour cells. Vascular cells (V) remain unstained. (**b**) BMPR-IB in a GBM showing positive immunostaining in the tumour. (**c**) ActR-I in a GBM showing positive staining in the tumour cells adjacent to blood vessels (V). (**d**) OP-1 immunostaining in a GBM. Moderate to strong staining is seen in glioma cells around vascular cells (V). (**e**) Strong BMPR-IA staining in U343 cells. (**f**) Weak staining of BMPR-IB in U343 cells. (**g**) No staining was seen for ActR-I in U1240 cells. (**h**) Distinct OP-1 immunostaining in U251 cells. (**i**) **a**–**d**, scale bar = 30 $\mu$m; **e**–**h**, scale bar = 15 $\mu$m.

using the SuperAnova program. The distribution of scaled results from the immunohistochemical analysis was normal. Independent categorical variables used were antigen and diagnostic groups. The difference between the three groups (GBM, low-grade and non-tumour) as to expression of BMP receptors was analysed for each antigen in tumour cells.

### Results

### *Expression of type I receptors for BMP and activin in glioma tissue*

The distribution of BMPR-IA, BMPR-IB and ActR-I in glioma tissues was examined using immunohistochemical techniques. Positive immunohistochemical staining for BMPR-IA was found in the cytoplasm of spindle-shaped tumour cells surrounding the blood vessels (Figure 1a, Table I). Strong immunoreactivity for BMPR-IB was found in the advancing edge of the cell-rich tumour areas (Figure 1b), in gemistocytic and other scattered tumour cells in the reactive areas. In contrast, astrocytoma, oligoastrocytoma and gliosis tissues showed weak or no staining (Table I). A distinct increase of BMPR-IB in tumour cells was observed in GBM compared with astrocytomas ($n=16$, $P<0.005$), and with gliosis ($n=13$, $P<0.001$). In contrast, BMPR-IA staining was moderate and ActR-I staining was only weakly expressed in some of the GBM tissues (Figure 1c). In gliotic tissues only weak expression of ActR-I or BMPR-I was seen in a few cases (Table I).

A weakly significant correlation of ActR-I staining with the degree of malignancy was found when all three groups were taken together ($n=21$, $P<0.05$). Very weak or no staining was detected in the areas of endothelial proliferation and extracellular matrix for BMPR-IB and BMPR-IA as well as for ActR-I. The control tissue sections, where primary antibodies were omitted, failed to stain positively, excluding the possibility of non-specific reactions by endogenous peroxidase, or detection systems. Specificities of the affinity-purified antibodies as well as the monoclonal OP-1 antibody were confirmed by nearly complete fading of the stainings when primary antibodies were blocked with the corresponding antigens.

### *Expression of OP-1 in glioblastoma tissue*

OP-1 was also detected in tissue sections of malignant glioma as revealed by staining with a monoclonal antibody against human OP-1. In all three glioblastoma multiforme tissues investigated, moderate staining was observed in the cytoplasm of glioma cells in high cell density areas (Figure 1d).

### *Expression of BMPR-Is and OP-1 in malignant glioma cell lines*

We have previously shown that [$^{125}$I]-OP1 binds to BMPR-IA, BMPR-IB and ActR-I on U1240MG cells (ten Dijke *et al.*, 1994*b*). Although it is difficult to know whether malignant glioma cell lines represent cells corresponding to GBM *in vivo*, expression of OP-1 and type I receptors in three human glioma cell lines was studied by immunocytochemistry. Using the monoclonal antibody against human OP-1, we found that OP-1 was expressed in all three glioma cell lines (Table II, Figure 1h). All these cell lines stained strongly positive by the BMPR-IA antibody (Table II, Figure 1e) and weakly by the BMPR-IB antibody (Figure 1f). However, no staining was detected by the ActR-I antibody (Figure 1g). Specificity of the antibodies to BMPR-IA and BMPR-IB was confirmed by quenching of the staining by preincubation of the antibodies with the corresponding peptides (not shown).

**Table II** Expression of OP-1 and its type I receptors in human malignant glioma cell lines

| *Cell line* | *BMPR-IA* | *BMPR-IB* | *ActR-I* | *OP-1* |
|---|---|---|---|---|
| U251MGsp | + | ± | – | + |
| U343MG | + + | ± | – | + |
| U1240MG | + | ± | – | + |

+ +, Moderate expression in most cells; +, low expression in most cells; ±, low expression in few cells; –, no expression. ActR-I, activin type I receptor; BMPR-IA, bone morphogenetic protein type IA receptor; BMPR-IB, bone morphogenetic protein type IB receptor.

### *Effects of OP-1 on the growth of malignant glioma cell lines*

OP-1 inhibited the growth of malignant glioma cell lines, U251MGsp, U343MGa and U1240MG as well as that of Mv1Lu, which was used as comparison; 20–40% inhibition of [$^3$H]thymidine incorporation was obtained at 10 nM OP-1 (Figure 2).

### Discussion

Members of the TGF-$\beta$ superfamily have been shown to inhibit the growth of various cell types (McCarthy and Bicknell, 1993; Wallen *et al.*, 1989; Matzuk *et al.*, 1992), however the effect of OP-1 on growth regulation has not been fully characterised. It has been reported that OP-1 stimulates the growth of osteoblasts (Sampath *et al.*, 1992), whereas it inhibits the growth of Mv1Lu cells (Yamashita *et al.*, 1995). The growth-inhibitory activities of OP-1 and activin A on Mv1Lu cells are approximately 100-fold lower than that of TGF-$\beta$1 (Yamashita *et al.*, 1995). Here, we show that OP-1 inhibits the growth of human malignant glioma cell lines. Interestingly, these cells are resistant to the growth-inhibitory action of TGF-$\beta$ (Yamada *et al.*, 1995), suggesting that the signalling pathways for growth inhibition are not entirely common for OP-1 and TGF-$\beta$1.

BMPR-IA is strongly expressed in glioma cell lines, whereas BMPR-IB is expressed at lower levels. In contrast, GBM tissues showed moderate to strong expression of BMPR-IB, and low to moderate expression of BMPR-IA. Expression of BMPR-Is was weaker in the low-grade astrocytoma, and very low in gliosis. Thus, a positive correlation between the malignancy grade of glioma and the expression of BMPR-IB was observed. On the other hand, the expression of ActR-I, which binds OP-1 but not BMP-4 (ten Dijke *et al.*, 1994*c*), was weak in malignant gliomas. These results suggest that glioma cells may be more responsive to the action of BMPs, compared with the cells in non-tumourous tissues and low-grade gliomas. Moreover, we showed that one of the ligands for BMPR-Is, i.e. OP-1, is

**Figure 2** Growth-inhibitory effects of OP-1 on Mv1Lu cells and human malignant glioma cell lines were determined by a [$^3$H]thymidine incorporation assay. Five experiments were done in triplicate; 20–40% inhibition of [$^3$H]thymidine incorporation was obtained at 10 nM OP-1. Mv1Lu (-●-); U251MGsp (-○-); U343MG (-□-); U1240MG (-△-).

also expressed in the glioma cells and tissues, suggesting that autocrine or paracrine control of cellular growth might take place in the glioma tissues. Northern blot analyses revealed that BMP-2 to -6 or OP-1 to -2 are not expressed in the normal brain (Sampath *et al.*, 1992). Other members in the BMP family, e.g. GDF-1 (Lee, 1991) and dorsalin-1 (Basler *et al.*, 1993), are expressed in the normal nervous systems.

The significance of the enhanced expression of OP-1 in the high-grade glioma for the malignant properties of the cells remains to be elucidated. Despite the fact that BMPs inhibit glioma cell growth, it is possible that they contribute to the malignant phenotype. Thus, BMPs act in the interaction of epithelial cells and mesenchyma (Lyons *et al.*, 1991), and stimulate chemotaxis (Cunningham *et al.*, 1992). They can also induce extracellular matrix and adhesion proteins (Vukicevic *et al.*, 1989; Paralkar *et al.*, 1992; Perides *et al.*, 1992, 1993) that are important in the invasive growth of tumour. Thus, it is possible that BMPs have stimulatory effects on glioma progression, directly or indirectly, through these or other mechanisms.

BMPR-II and ActR-IIs, which can function as type II receptors for BMPs, are expressed in brain (Mathews and Vale, 1991; Rozenzweig *et al.*, 1995). It remains to be seen whether they are expressed in glioma. Elucidation of the functional roles of BMPs in glioma awaits further studies.

**Acknowledgements**
We thank Drs Monica Nistér and Bengt Westermark at the Department of Pathology for obtaining glioma cell lines and tissues, and Ms Aive Åhgren for excellent technical assistance. This study was partially supported by the Fredrik and Ingrid Thuring Foundation.

### References

Basler K, Edlund T, Jessel TM and Yamada T. (1993). Control of cell pattern in the neural tube: regulation of cell differentiation by dorsalin-1, a novel TGF$\beta$ family member. *Cell*, **73**, 687–702.

Celeste AJ, Iannazzi JA, Taylor RC Hewick RM, Rosen V, Wang EA and Wozney JM. (1990). Identification of transforming growth factor $\beta$ family members present in bone-inductive protein purified from bovine bone. *Proc. Natl Acad. Sci. USA*, **87**, 9843–9847.

Collins UP and James CD. (1993). Genes and chromosomal alterations associated with the development of human gliomas. *FASEB J.*, **7**, 926–930.

Cunningham NS, Paralkar V and Reddi AH. (1992). Osteogenin and recombinant bone morphogenetic protein 2B are chemotactic for human monocytes and stimulate transforming growth factor $\beta$1 mRNA expression. *Proc. Natl Acad. Sci. USA*, **89**, 11740–11744.

Dewulf N, Verschueren K, Lonnoy O, Morén A, Grimsby S, Spiegle KV, Miyazono K, Huylebroeck D and ten Dijke P. (1995). Distinct spatial and temporal expression patterns of two type I receptors for bone morphogenetic proteins during mouse embryogenesis. *Endocrinology*, **136**, 2652–2663.

Hermanson M, Funa K, Hartman M, Claesson-Welsh L, Heldin C-H, Westermark B and Nistér. (1992). Platelet-derived growth factor and its receptors in human glioma tissue: Expression of messenger RNA and protein suggests the presence of autocrine and paracrine loops. *Cancer Res.*, **52**, 3213–3219.

Kingsley DM. (1994*a*). What do BMPs do in mammals? Clues from the mouse short-ear mutation. *Trends Genet.*, **10**, 16–21.

Kingsley DM. (1994*b*). The TGF-$\beta$ superfamily: new members, new receptors, and new genetic tests of function in different organisms. *Genes Dev.*, **8**, 133–146.

Kleihues P, Burger PC and Scheithauer BW. (1993). Histological typing of tumours of the central nervous system. In *International Histological Classification of Tumours*. World Health Organization: Geneva.

Lee S-J. (1991). Expression of growth/differentiation factor 1 in the nervous system: Conservation of bicistronic structure. *Proc. Natl Acad. Sci. USA*, **88**, 4250–4254.

Libermann TA, Nusbaum HR, Razon N, Kris R, Lax I, Soreq H, Whittle N, Waterfield MD, Ullrich A and Schlessinger J. (1985). Amplification, enhanced expression and possible rearrangement of EGF-receptor gene in primary human brain tumours of glioma origin. *Nature*, **313**, 144–147.

Lyons KM, Jones CM and Hogan BLM. (1991). The *DVR* gene family in embryonic development. *Trends Genet.*, **7**, 408–412.

Massagué J, Attisano L and Wrana JL. (1994). The TGF-$\beta$ family and its composite receptors. *Trends Cell Biol.*, **4**, 172–178.

McCarthy SA and Bicknell R. (1993). Inhibition of vascular endothelial cell growth by activin-A. *J. Biol. Chem.*, **268**, 23066–23071.

Mathews LS and Vale WW. (1991). Expression cloning of an activin receptor, a predicted transmembrane serine kinase. *Cell*, **65**, 975–982.

Matzuk MM, Finegold MJ, Su J-GJ, Hsueh AJW and Bradley A. (1992). $\alpha$-Inhibin is a tumour-suppressor gene with gonadal specificity in mice. *Nature*, **360**, 313–319.

Nigro JM, Baker SJ, Preisinger AC, Jessup JM, Hostetter R, Cleary K, Bigner SH, Davidson N, Baylin S, Devilee P, Glover T, Collins FS, Weston A, Modali R, Harris CC and Vogelstein B. (1989). Mutations in the p53 gene occur in diverse human tumour types. *Nature*, **342**, 705–708.

Nistér M, Claesson-Welsh L, Eriksson A, Heldin C-H and Westermark B. (1991). Differential expression of platelet-derived growth factor receptors in human malignant glioma cell lines. *J. Biol. Chem.*, **266**, 16755–16763.

Özkaynak E, Rueger DC, Drier EA, Corbett C, Ridge RJ, Sampath TK and Oppermann H. (1990). OP-1 cDNA encodes an osteogenic protein in the TGF-$\beta$ family. *EMBO J.*, **9**, 2085–2093.

Özkaynak E, Schnegelsberg PNJ, Jin DF, Clifford GM, Warren FD, Drier EA and Oppermann H. (1992). Osteogenic protein-2: a new member of the transforming growth factor-$\beta$ superfamily expressed early in embryogenesis. *J. Biol. Chem.*, **267**, 25220–25227.

Paralkar VM, Weeks BS, Yu YM, Kleinman HK and Reddi AH. (1992). Recombinant human bone morphogenetic protein 2B stimulates PC12 cell differentiation: potentiation and binding to type IV collagen. *J. Cell Biol.*, **119**, 1721–1728.

Perides G, Safran RM, Rueger DC and Charness ME. (1992). Induction of the neural cell adhesion molecule and neuronal aggregation by osteogenic protein 1. *Proc. Natl Acad. Sci. USA*, **89**, 10326–10330.

Perides G, Hu G, Rueger DC and Charness ME. (1993). Osteogenic protein-1 regulates L1 and neural cell adhesion molecule gene expression in neural cells. *J. Biol. Chem.*, **268**, 25197–25205.

Reddi AH. (1992). Regulation of cartilage and bone differentiation by bone morphogenetic proteins. *Curr. Opin. Cell Biol.*, **4**, 850–855.

Rozenzweig BL, Imamura T, Okadome T, Cox GN, Yamashita H, ten Dijke P, Heldin C-H and Miyazono K. (1995). Cloning and characterization of a human type II receptor for bone morphogenetic proteins. *Proc. Natl Acad. Sci. USA*, **92**, 7632–7636.

Sampath TK, Maliakal JC, Hauschka PV, Jones WK, Sasak HM, Tucker RF, White KH, Coughlin JE, Tucker MM, Pang RH, Corbett C, Özkaynak E, Oppermann H and Rueger DC. (1992). Recombinant human osteogenic protein-1 (hOP-1) induces new bone formation *in vivo* with a specific activity comparable with natural bovine osteogenic protein and stimulates osteoblast proliferation and differentiation *in vitro*. *J. Biol. Chem.*, **267**, 20352–20362.

Storm EE, Huynh TV, Copeland NG, Jenkins NA, Kingsley DM and Lee S-J. (1994). Limb alterations in *Brachypodism* mice due to mutations in a new member of the TGF$\beta$ superfamily. *Nature*, **368**, 639–643.

ten Dijke P, Ichijo H, Franzén P, Schulz P, Saras J, Toyoshima H, Heldin C-H, and Miyazono K. (1993). Activin receptor-like kinases; A novel subclass of cell-surface receptors with predicted serine/threonine kinase activity. *Oncogene*, **8**, 2879–2887.

TEN DIJKE P, FRANZÉN P, YAMASHITA H, ICHIJO H, HELDIN C-H AND MIYAZONO K. (1994*a*). Serine/threonine kinase receptors. *Prog. Growth Factor Res.*, **6,** 55–72.

TEN DIJKE P, YAMASHITA H, SAMPATH TK, REDDI AH, ESTEVEZ M, RIDDLE DL, ICHIJO H, HELDIN C-H AND MIYAZONO K. (1994*b*). Characterization of type I receptors for transforming growth factor-$\beta$ and activin. *Science*, **264,** 101–104.

TEN DIJKE P, YAMASHITA H, ICHIJO H, FRANZÉN P, LAIHO M, MIYAZONO K AND HELDIN C-H. (1994*c*). Identification of type I receptors for osteogenic protein-1 and bone morphogenetic protein-4. *J. Biol. Chem.*, **269,** 16985–16988.

VENTER DJ, BEVAN KL, LUDWIG RL, RILEY TE, JAT PS, THOMAS DG AND NOBLE MD. (1991). Retinoblastoma gene deletions in human glioblastomas. *Oncogene*, **6,** 445–448.

VERSCHUEREN K, DEWULF N, GOUMANS M-J, LONNOY O, FEIJEN A, GRIMSBY S, VANDE SPIEGLE K, TEN DIJKE P, MORÉN A, VANSCHEEUWIJCK P, HELDIN C-H, MIYAZONO K, MUMMERY C, VAN DEN EIJNDEN-VAN RAAIJ J AND HUYLEBROECK D. (1995). Expression of type I and type IB receptors for activin in midgestation mouse embryos suggests distinct functions in organogenesis. *Mech. of Dev.*, **52,** 109–123.

VUKICEVIC S, LUYTEN FP AND REDDI AH. (1989). Stimulation of the expression of osteogenic and chondrogenic phenotypes *in vitro* by osteogenin. *Proc. Natl Acad. Sci. USA*, **86,** 8793–8797.

VUKICEVIC S, LATIN V, CHEN P, BATORSKY R, REDDI AH AND SAMPATH TK. (1994). Localization of osteogenic protein-1 (bone morphogenetic protein-7) during human embryonic development: high affinity binding to basement membrane. *Biochem. Biophys. Res. Commun.*, **198,** 693–700.

WALLEN JW, CATE RL, KIEFER DM, RIEMEN MW, MARTINEZ D, HOFFMAN RM, DONAHOE PK, VON HOFF DD, PEPINSKY B AND OLIFF A. (1989). Minimal antiproliferative effect of recombinant Mullerian inhibiting substance on gynecological tumour cell lines and tumour explants. *Cancer Res.*, **49,** 2005–2011.

WALTENBERGER J, LUNDIN L, ÖBERG K, WILANDER E, MIYAZONO K, HELDIN C-H AND FUNA K. (1993*a*). Involvement of transforming growth factor-$\beta$ in the formation of fibrotic lesions in carcinoid heart disease. *Am. J. Pathol.*, **142,** 71–78.

WALTENBERGER J, WANDERS A, FELLSTRÖM B, MIYAZONO K, HELDIN C-H AND FUNA K. (1993*b*). Induction of transforming growth factor-$\beta$ during cardiac allograft rejection. *J. Immunol.*, **151,** 1147–1157.

WONG AJ, BIGNER SH, BIGNER DD, KINZLER KW, HAMILTON SR AND VOGELSTEIN B. (1987). Increased expression of the epidermal growth factor receptor gene in malignant gliomas is invariably associated with gene amplification. *Proc. Natl Acad. Sci. USA*, **84,** 6899–6903.

WOZNEY JM. (1989). Bone morphogenetic proteins. *Prog. Growth Factor Res.*, **1,** 267–280.

WOZNEY JM, ROSEN V, CELESTE AJ, MITSOCK LM, WHITTERS MJ, KRIZ RW, HEWICK RM AND WANG EA. (1988). Novel regulators of bone formation: molecular clones and activities. *Science*, **242,** 1528–1534.

WRANA JL, ATTISANO L, WIESER R, VENTURA F AND MASSAGUÉ J. (1994). Mechanism of activation of the TGF-$\beta$ receptor. *Nature*, **370,** 341–347.

YAMADA N, KATO M, YAMASHITA H, NISTÉR M, MIYAZONO K, HELDIN C-H AND FUNA K. (1995). Enhanced expression of transforming growth factor-$\beta$ and its type I and type II receptors in human glioblastoma multiforme. *Int. J. Cancer*, **62,** 1–7.

YAMASHITA H, TEN DIJKE P, HUYLEBROECK D, SAMPATH TK, ANDRIES M, SMITH JC, HELDIN C-H AND MIYAZONO K. (1995). Osteogenic protein-1 binds to activin type II receptor and induces certain activin-like effects. *J. Cell Biol.*, **130,** 217–226.